# Production of hydrogen peroxide in an intra-meander hyporheic zone at East River, Colorado

**DOI:** 10.1038/s41598-021-04171-1

**Published:** 2022-01-13

**Authors:** Xiu Yuan, Tongxu Liu, Patricia Fox, Amrita Bhattacharyya, Dipankar Dwivedi, Kenneth H. Williams, James A. Davis, T. David Waite, Peter S. Nico

**Affiliations:** 1 Guangdong Key Laboratory of Integrated Agro-Environmental Pollution Control and Management, Institute of Eco-Environmental and Soil Sciences, Guangdong Academy of Sciences, Guangzhou, 510650 China; 2 Earth and Environmental Sciences, Lawrence Berkeley National Laboratory, 1 Cyclotron Road, Berkeley, CA 94720 USA; 3 Water Research Centre, School of Civil and Environmental Engineering, The University of New South Wales, Sydney, NSW 2052 Australia

**Keywords:** Biogeochemistry, Environmental sciences

## Abstract

The traditionally held assumption that photo-dependent processes are the predominant source of H_2_O_2_ in natural waters has been recently questioned by an increrasing body of evidence showing the ubiquitiousness of H_2_O_2_ in dark water bodies and in groundwater. In this study, we conducted field measurement of H_2_O_2_ in an intra-meander hyporheic zone and in surface water at East River, CO. On-site detection using a sensitive chemiluminescence method suggests H_2_O_2_ concentrations in groundwater ranging from 6 nM (at the most reduced region) to ~ 80 nM (in a locally oxygen-rich area) along the intra-meander transect with a maxima of 186 nM detected in the surface water in an early afternoon, lagging the maximum solar irradiance by ∼ 1.5 h. Our results suggest that the dark profile of H_2_O_2_ in the hyporheic zone is closely correlated to local redox gradients, indicating that interactions between various redox sensitive elements could play an essential role. Due to its transient nature, the widespread presence of H_2_O_2_ in the hyporheic zone indicates the existence of a sustained balance between H_2_O_2_ production and consumption, which potentially involves a relatively rapid succession of various biogeochemically important processes (such as organic matter turnover, metal cycling and contaminant mobilization). More importantly, this study confirmed the occurrence of reactive oxygen species at a subsurface redox transition zone and further support our understanding of redox boundaries on reactive oxygen species generation and as key locations of biogeochemical activity.

## Introduction

Reactive oxygen species (ROS), such as superoxide (O_2_^**·**−^), hydrogen peroxide (H_2_O_2_) and hydroxyl radical (HO^**·**^), are oxygen-containing reactive molecules with considerable environmental importance due to their high reactivity in mediating redox transformations^1,2^. Extensive studies have shown that ROS are involved in various biogeochemical and ecologically significant processes, including nutrient cycling^3,4^, contaminant transformation^5^, oxidative stress and intracellular signalling in organisms^6–8^, as well as the establishment of microbial symbioses^9,10^. In addition, recent increasing recognition of the significant roles of ROS in soil carbon remineralisation^11,12^ and metal bioavailability^3^ urges a more comprehensive understanding of ROS generation and transformation in natural environments.

The dominant source of ROS in natural aquatic environments has long been attributed to photo-dependent processes. For example, photo-oxidation of chromophoric dissolved organic matter (CDOM) is generally considered the predominant source of H_2_O_2_ (the longest-lived ROS) in surface waters, with a number of studies reporting the occurrence of H_2_O_2_ in a wide range of natural waters, including oceans^13–16^, lakes^17–19^, coastal and estuarine waters^20,21^, freshwater^22–24^, and geothermal waters^25,26^. However, this commonly held assumption has been recently questioned by an increasing body of evidence demonstrating ROS production under dark conditions, either of biological and/or chemical origins^27–30^. To be more specific, while Zhang et al.^27^ reported the widespread occurrence of ROS (O_2_^**·**−^ and H_2_O_2_) in dark brackish and freshwater environments and attributed such dark profiles (at comparable concentrations as in sunlit waters) to biological contributions, Page et al.^28^ and Tong et al.^31^ detected extensive abiotic ROS flux from oxygenation of reduced humic acids and subsurface sediments under dark conditions, respectively. Further, our recent field study revealed light-independent generation of H_2_O_2_ in groundwater of an alluvial aquifer adjacent to the Colorado River near Rifle, CO and demonstrated that fluctuating redox conditions created at oxic/anoxic interfaces are likely to be the key locations for ROS generation, thereby expanding the relevance of these reactive molecules to the subsurface domain, possibly the least understood component of biogeochemical cycles^30^.

Hyporheic exchange refers to the bi-directional exchange of mass, energy and living organisms between rivers and the shallow subsurface waters, leading to interactions between nutrient-rich groundwater and oxygen-rich river water, along with the formation of distinct redox and hydrodynamic gradients^32,33^. Hyporheic zone has been identified as a critical component of river systems for decades due to its wide reaching consequences for water quality and stream ecology^34–36^. For example, the constant mixing of surface water that typically has abundant oxygen content and high exposure to light, with groundwater that is more reduced and devoid of light but usually rich in nutrients renders the hyporheic zone highly redox-dynamic and ideally suited for ROS production. More importantly, such intensive mixing between waters with largely different chemical compositions facilitates microbial activity and diversity in a river system, not only leading to further generation of ROS via the biological pathway, but also resulting impact on the stream metabolism, carbon and nutrient cycling^33,37–40^. The lack of field-deployable, real-time measurement of ROS in such dynamic redox regimes has left a significant gap in understanding the ROS biogeochemical cycle and hindered our complete appreciation of their importance in the ecosystem as a whole. Therefore, as an extension of our field study on H_2_O_2_ at the Rifle site^30^, field measurements of H_2_O_2_ were conducted at the intra-meander region of the East River floodplain in Colorado, with the aim of further exploring the occurrence of ROS in a typical hyporheic zone.

## Materials and methods

### Reagents

Analytical grade chemicals were purchased from Sigma-Aldrich or VWR International (or as otherwise stated) and used without further refinement. Acridinium ester (AE, 10-methyl-9-(*p*-formylphenyl)acridinium carboxylate trifluoromethanesulfonate) for measurement of H_2_O_2_ was purchased from Cayman Chemical (Michigan, USA). AE reagent of 5.0 µM at pH 3.0 was prepared each sampling day by dilution of a refrigerated AE stock solution (250 µM, pH 3.0) by adding 1.0 mM phosphate buffer (pH 3.0). H_2_O_2_ (30%, ultra-high purity) was purchased from BDH Chemicals and calibrated spectrophotometrically using a UV–Vis spectrophotometer^41^. 0.1 M carbonate buffer of pH 11.3 was prepared weekly and other stock solutions were refrigerated at 4 °C in the dark.

### Field information and experimental measurement

#### Field site and water sampling

**Figure 1:**
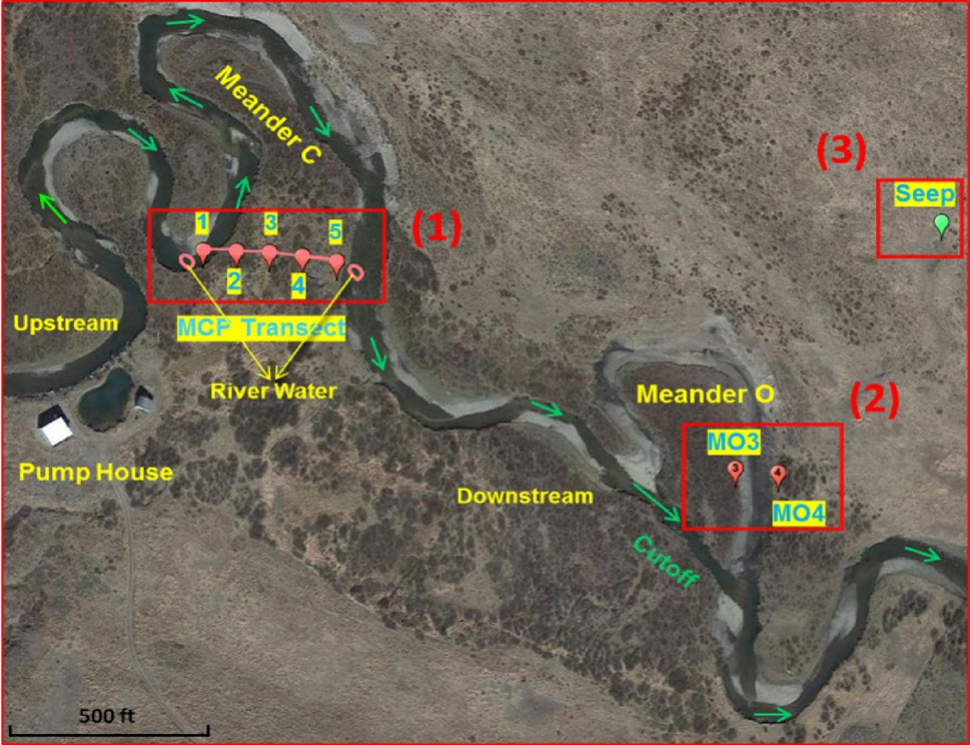
Sampling location for H_2_O_2_ detection in the East River floodplain, including three groups of sampling wells: (1) The MCP transect within the intra-meander hyporheic zone, including five piezometric observation wells (MCP1 to MCP5); (2) Two sampling wells (MO3 and MO4) close to the new river channel of Meander O (the “neck cutoff”); (3) One sampling well (Seep) at the Mancos Shale seep (approximately 150 m uphill from the oxbow lake of Meander O). Green arrows indicate the flow direction in the river. This image was acquired and modified from *Google Earth*, earth.google.com/web/.

Field measurements of H_2_O_2_ were conducted in middle July 2016 at the East River floodplain, located in a high elevation catchment in southwestern Colorado (between 38.8° to 38.9° N and 106.8° to 106.9° W) with rolling-to-mountainous topography. The floodplain includes multiple river meanders that extend over a distance of 11 km (Fig. 1). The site encompasses the drainages of the East River and Copper Creek with the stream flow fed predominantly by snowmelt in late spring to early summer and mid- to late-summer monsoonal rainfall triggering rapid but punctuated increases in flow. The area experiences an average temperature ranging from a low of − 8 °C in January to a high of 35 °C in July. More site information can be found in the recent study by Dwivedi et al.^32^ Because of the inherent spatial heterogeneity in a floodplain environment, we investigated a typical transect of Meander C of the East River site that is approximately parallel to the hyporheic flow field, labeled as MCP on Fig. 1. Specifically, the MCP transect includes five piezometric observation wells for geochemical samples (Wells MCP1 to MCP5) with these wells and transect accordingly referred to as Meander C Piezometric (MCP) wells and MCP transect (Fig. 1). Stainless steel drive-point piezometers were installed to achieve a sampling depth of 1.85 m below ground surface. Groundwater was pumped from each piezometer using a peristaltic pump and sampled after purging a volume of water equivalent to three times the water standing in each piezometer. H_2_O_2_ detection was performed both in groundwater and in the surface river water at meander C. Groundwater was peristaltically pumped through opaque flexible tubing into an amber high density polyethylene (HDPE) bottle with no head space and was analysed within 4–6 min after collection. Filtration of the groundwater by 0.22 µm filters made no difference on the final concentration of H_2_O_2_, therefore no filtration was done to minimize the sampling time. Unfiltered groundwater samples were collected for pH, electrical conductivity (EC) (data published in Dwivedi et al.^32^, not included in this study), oxidation–reduction potential (ORP) and dissolved oxygen in the field. Filtered (0.45 m PVDF syringe filters) samples were collected for in-field ferrous ion (Fe(II)) measurement and laboratory analysis of metals and dissolved organic and inorganic carbon. Samples for elemental analysis and carbon measurements were collected and acidified to pH 3 with HCl (high purity, 30% w/v; Sigma) and kept cool or refrigerated until shipped to the laboratory in Berkeley, CA.

#### H_2_O_2_ and other element analysis

H_2_O_2_ concentrations were determined by the chemiluminescence method^42,43^ using a flow injection FeLume system (Waterville Analytical, Waterville, ME) with detailed information reported in our previous study^30^. To avoid any possible interference caused by additional interactions between ferrous iron and O_2_ under alkaline conditions^43^, ferrozine solution (3-(2-pyridyl)-5,6-diphenyl-1,2,4-triazine-4′,4′′-disulfonic acid sodium salt, Sigma) was added to bind all the ferrous species before the sample entered the flow cell located at the photo multiplier tube interface where it mixed with a carbonate (pH 11.3) buffer to initiate the chemiluminescence-generating reaction. Dissolved oxygen (DO) and Fe(II) were measured colorimetrically in the field using a portable spectrophotometer and CHEMetrics vacu-vials (chemets). DO was measured either with the Rhodazine D method^44^ for DO < 1 ppm (Kit No.: K-7553) or Indigo Carmine method^45^ for 1–15 ppm (Kit No.: K-7513) with accuracy ranging from ± 0.025 to ± 0.080 ppm and ± 0.6 to ± 1.1 ppm, respectively. Fe(II) was measured using the phenanthroline method (Kit No.: K-6203, 0–6 ppm) with accuracy ranging from ± 0.08 to ± 0.045 ppm. Dissolved organic carbon (DOC) concentrations were determined by the non-purgeable organic carbon method on a Shimadzu TOC-V analyzer. Samples were acidified with HCl and purged with N_2_ in order to remove inorganic carbon prior to analysis.

## Results and discussion

### H_2_O_2_ profiles measured at the Meander C transect

**Figure 2:**
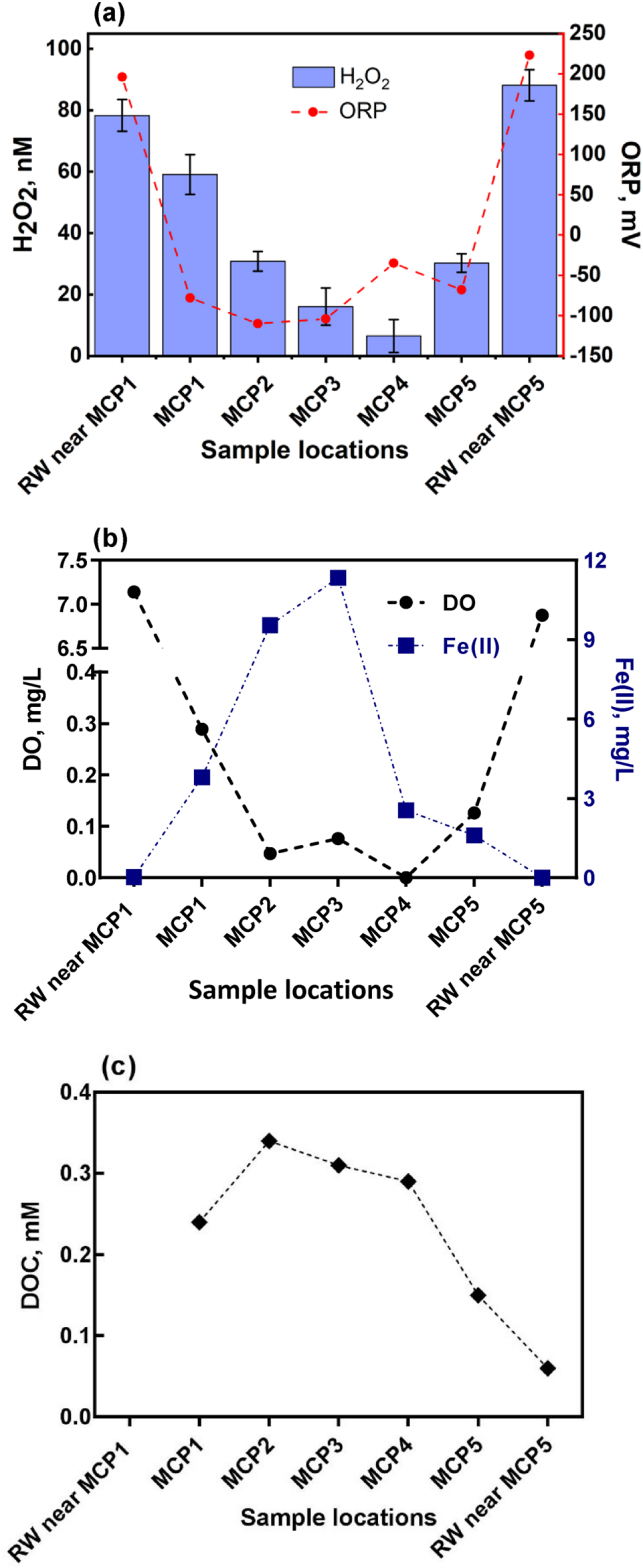
(**a**) H_2_O_2_ and ORP profile in the hyporheic zone at Meander C Transect. The first and last columns are results from surface water samples taken at the river adjacent to MCP1 (RW near MCP1) and MCP5 (RW near MCP5), respectively. Error bars are the standard errors from triplicate measurements. (**b**) Profiles of DO (left Y axis with two segments) and Fe(II) (right Y axis) in the hyporheic zone and surface river water at Meander C transect. (**c**) DOC concentrations across the hyporheic zone and in river water near MCP5.

The East River floodplain, as shown in Fig. 1, includes multiple active river meanders, fresh oxbow lakes and older oxbow deposits, within which pronounced redox gradients have been observed during preliminary work^32,46^. Among all the active meanders at the site, the one designated meander C in Fig. 1 is typical in many river meanders with hyporheic flowpaths cutting across large sections of the meander, leading to the potential of substantial sinuosity-driven hyporheic exchange and formation of sharp redox gradients that favor the generation of ROS^47^. Therefore, H_2_O_2_ measurements were first conducted at transect of Meander C that is approximately parallel to the hyporheic flow field where seasonal fluxes are predicted to occur^32,36^ (labeled as MCP in Fig. 1). In order to obtain an estimation of the differences in H_2_O_2_ content in the river water and in the hyporheic zone, measurement of H_2_O_2_, oxidation–reduction potential (ORP), dissolved oxygen (DO), Fe(II) and dissolved organic carbon (DOC) was also conducted in surface river water adjacent to the bank, with results shown in Fig. 2. First of all, despite the prevailing reducing conditions at MCP wells, concentrations of H_2_O_2_ were significantly above the detection limit (1.0 nM)^30^ in all waters sampled across the Meander C transect with a maximum of 88 nM found in the surface water and a minimum of 6.4 nM detected in the aphotic zone located in the middle of the intra-meander (MCP4, likely the most reduced region across the transect). Our detected H_2_O_2_ profile in groundwater agrees with another field study conducted at Sand Ridge site, Illinois, which reported persistent detection of H_2_O_2_ at nanomolar levels in groundwater over a 2-year period^48^. Figure 2 clearly demonstrates that H_2_O_2_ pattern closely follows the local redox gradient, with relatively abundant H_2_O_2_ generated in areas with more DO available and less H_2_O_2_ occurring in regions under severely reducing conditions, as indicated by low ORP and high Fe(II) concentrations (Fig. 2b). As suggested in our previous study^30^, considering the slow groundwater flux velocity along the meander (< 10^–5^ m/s)^32^, H_2_O_2_ observed in the hyporheic zone was presumably actively produced in the vicinity of the sampling location rather than from H_2_O_2_ transported downstream from the river water. To be more specific, a rough calculation from the data reported in Moffett and Zajiriou^49^ provided a H_2_O_2_ decay rate of 0.055 h^−1^ for unfiltered seawater (Table 1 in Moffett and Zajiriou^49^), indicating a half-life of 12 h for our detected H_2_O_2_ (Fig. 2a). At the same time, the 78 nM H_2_O_2_ observed in the river water near MCP1 will decrease to ~ 1 nM after three days. For the same meander, the groundwater velocities reported in Dwivedi et al.^32^ are in the range of 1.25 × 10^–5^ to 5.09 × 10^–6^ m/s in the top 2 m, which suggested that the residence time of groundwater along the MCP transect was on the order of 1 to 2 months. Therefore, peroxide in river water would be all consumed before reaching the sampling points and H_2_O_2_ transport within 2 days of the sampling time will not contribute to our data at any significant level. Moreover, fresh water has been reported to have higher H_2_O_2_ decay rates, with a range of 0.11–8.9 h^−1^ reported by Bond et al.^40^ based on previous investigations, suggesting that our estimation for H_2_O_2_ decay here is highly conservative, as H_2_O_2_ consumption in hyporheic zone will be much faster due to greater microbial activity.

The absence of detectable dissolved oxygen at MCP4 (lower than the detection limit of 20 µg/L) did not negate the occurrence of H_2_O_2_ completely, though the distinguishable rebound of ORP value at this location (Fig. 2a) suggested a slight variation in the “bulk” redox condition of local water, possibly resulting from the existence of other oxidants (nitrate, iron, and sulfate et al.). Similar findings were evident in our previous study at the Rifle, CO field site where measureable H_2_O_2_ was discovered in regions of the aquifer characterized as strongly O_2_-deficient^30^. Recent studies by Zhang et al.^29^ and Borda et al.^50^ proposed that H_2_O_2_ production resulted from the combination of two HO^**·**^ formed by the oxidation of water at sulfur-deficient sites on pyrite surfaces (Eq. 1).

1 $$\equiv {\text{Fe(III)}} + {\text{H}}_{{2}} {\text{O}}_{{({\text{ads}})}} \to \, \equiv {\text{Fe(II)}} + {\text{ HO}}_{{({\text{ads}})}}^{ \cdot } + {\text{H}}^{ + }$$

These studies were undertaken under anoxic conditions, demonstrating that O_2_ is not necessarily a prerequisite for ROS formation under such circumstances. While speculative at this stage, such a mechanism could account for the observed H_2_O_2_ at MCP4 considering that the East River valley in the study area is comprised of Mancos Shale bedrock formed by an agglomeration sequence of heterogeneous marine black shale with regions of elevated metal, metalloid, and pyrite content^46,51^. This is further supported in a recent study by Dwivedi et al.^32^ revealing that pyrite is one of the major reactive minerals in river sediments collected from the MCP transect.

It is not unexpected that Fe(II) profile observed in the hyporheic zone at Meander C is roughly in an opposite pattern to that of DO (or at least for results from wells MCP1 to MCP3), as shown in Fig. 2b. Oxygen was consumed rapidly when moving from the river bank to the center of the meander, with a concomitant increase in Fe(II) concentration, suggesting that when oxygen content decreased to a certain level, other electron acceptors that are available, such as nitrate, iron, and sulfate, could also be reduced. Dwivedi et al.^32^ also reported that the redox zonation along the MCP transect was highly dynamic and predominantly driven by groundwater velocities resulting from river-stage fluctuations. The presence of elevated Fe(II) at well MCP3 indicated that the groundwater along the meander centerline was probably segregated and not actively receiving oxygenated recharge. The oxidation of reduced transition metals, such as Fe(II) (in both complexed and uncomplexed forms), by oxygen has long been reported to result in the formation of O_2_^**·**−^ as a by-product and subsequent formation of H_2_O_2_ by its disproportionation or further reaction with reduced metals or organics (Eqs. 2–4)^52–55^. While the di-radical reaction (Eq. 3) is less important considering the very low concentration of O_2_^**·**−^, its further interactions with reduced metals (Eq. 4) and/or organics (such as hydroquinones)^55,56^ could play a significant role in H_2_O_2_ generation. Therefore, constant hyporheic exchange could facilitate the generation of ROS by the extensive intrusion of oxygen-rich river water to the porous, nutrient-rich sediment, as shown in a recent study by Murphy et al.^11^, in which the transformation of H_2_O_2_ was observed when iron(II) monosulfide was exposed to oxic conditions caused by tidal cycling.

2 $${\text{Fe(II) + O}}_{{2}} { } \to {\text{ Fe(III) + O}}_{2}^{ \cdot - } ,$$

3 $${\text{O}}_{{2}}^{\cdot - } {\text{ + O}}_{2}^{\cdot - } {\text{ + 2H}}^{ + } { } \to {\text{ H}}_{{2}} {\text{O}}_{{2}} {\text{ + O}}_{{2}} ,$$

4 $${\text{Fe(II) + O}}_{2}^{\cdot - } \to {\text{ H}}_{{2}} {\text{O}}_{{2}} {\text{ + Fe(III)}}{.}$$

It is also noteworthy from Fig. 2 that even though the concentrations of both DO and Fe(II) are more elevated at MCP3 than that at MCP2, a lower H_2_O_2_ content was detected in waters sampled at MCP3, indicating that the magnitude of H_2_O_2_ presence at such locations is likely to be co-mediated by mechanisms other than simply the interaction between DO and Fe(II). In fact, the co-occurrence of H_2_O_2_ and Fe(II) presents the possibility of Fenton-type reactions occurring, which have been generally considered to promote H_2_O_2_ decomposition and generation of more reactive oxidants, including HO^**·**^ (an indiscriminate oxidant that reacts rapidly with organic carbon and other recalcitrant compounds, Eq. 5) and high valent Fe species^57–59^. Similar opposite patterns of Fe(II) and dissolved oxygen was also observed in a field study at Illinois by Barcelona, et al. between 1984 to 1986^48^, which indicated that such chemical gradients were very stable during their 2-year observation period regardless of the changes in reginal water levels at a rate of ~ 0.4 m/year.

5 $${\text{Fe(II)}} + {\text{H}}_{{2}} {\text{O}}_{2} \to {\text{Fe(III)}} + {\text{ HO}}^{ \cdot } + {\text{OH}}^{ - } .$$

Futher, the organic carbon content presented across this hyporheic transect, as shown in Fig. 2c with an opposite pattern to that of H_2_O_2_, could also contribute to the evolution of ROS. Page et al.^12^ observed that more HO^**·**^ was generated in more reduced, iron and organic carbon rich waters and proposed that such dark HO^**·**^ formation came from the oxidation of ferrous iron (through dark Fenton reactions) and reduced organic carbon by oxygen. This is in agreement with our observation that less H_2_O_2_ was presented in regions with higher organic carbon content, indicating that more H_2_O_2_ were involved in reactions with soil carbon to produce other more reactive species that can further contribute to carbon cycling and pollutant degradation^56,60^. Recently Tong et al.^31^ reported an abundant generation of HO^**·**^ (with H_2_O_2_ acting as the intermediate) on oxygenation of various subsurface sediments sampled from a variety of typical redox-fluctuating subsurface environments and found that Fe(II)-containing minerals, particularly phyllosilicates, are the predominant contributor to HO^**·**^ formation. Consequently, it is clear that these various forms of Fe(II) species not only contribute to H_2_O_2_ formation, but are also involved in subsequent transformation of H_2_O_2_ to HO^**·**^, potentially resulting in mineralization of organic carbon to CO_2_ at rates that are much higher than those estimated for oxygenation and photolysis of surface waters and of the same order of magnitude as that produced from soil respiration^31,61–64^. Since soils contain more than three times as much carbon as either the atmosphere or terrestrial vegetation, changes in this major pool of organic carbon could have huge impacts on the global carbon budget^65–67^, despite the fact that processes affecting carbon migration and transformations in the subsurface domain remain the least understood component of the global carbon cycle.

The extremely low concentration of Fe(II) in surface water near either MCP1 or MCP5 in Fig. 2b indicates that it is unlikely to be an important participant in generation and/or consumption of H_2_O_2_ in such oxygenated system. Rather, solar radiation-driven photochemical and phototrophic processes are generally considered the predominant source of H_2_O_2_ in such surface sunlit waters. Nevertheless, despite the aforementioned various possible chemical mechanisms for H_2_O_2_ generation in the intra-meander hyporheic zone, biological production is another essential contributor, and could become the dominant source under certain conditions. For example, Sutherland et al.^68^ recently reported that light-independent superoxide (the potential precursor of H_2_O_2_, Eq. 2) concentrations in marine environment exhibited significant spatial heterogeneity (10–2000 pM) and microorganisms can account for a significant fraction (84% and 40% for on and off-shelf seawater, respectively) of such ROS generation. Another study on dark transformation of H_2_O_2_ in varying freshwater settings by Marsico et al.^69^ reported a wide range of production (3–259 nM h^–1^) and decay rates (0.02–8.87 h^–1^), and suggested that microbes are the predominant contributor to both H_2_O_2_ generation and consumption in the system. Although investigations on the biotic community that is actively contributing to the observed H_2_O_2_ patterns in both groundwater and surface water at this transect is beyond the scope of this study, it is worth mentioning that there is increasing evidence demonstrating the involvement of various groups of microorganisms in extracellular ROS production, including fungi, various species of phytoplankton and microalgae, symbionts in corals, and heterotrophic bacteria^6,27,70^.

### Stability of hyporheic H_2_O_2_ concentrations compared to surface water

**Figure 3:**
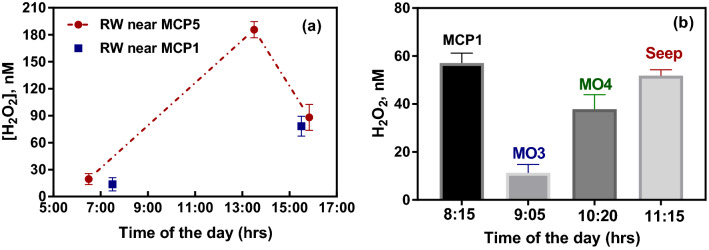
(**a**) Diel changes of H_2_O_2_ in surface river waters adjacent to MCP5 (red circles) and MCP1 (blue squares), with dashed line shown just as connections between data points; (**b**) H_2_O_2_ concentrations in groundwater measured at other locations of the East River site. Error bars are the standard errors from triplicate measurements.

In order to reach a better understanding on whether hyporheic ROS profiles were driven by changes in surface water ROS concentrations, measurments were also conducted in river water close to the bank at different times of the day, with results shown in Fig. 3. It is apparent in Fig. 3a that H_2_O_2_ in the surface river water exhibited a pronounced diel variation, in line with the importance of light-mediated pathways (photochemical and/or phototrophic) on ROS generation and consumption^27,71–73^. Take the river water adjacent to MCP5 as an example, H_2_O_2_ concentrations increased rapidly from the early morning (~ 20 nM at 6:30 a.m.) to 1:30 pm (a maximum detected concentration of 186 nM) and then decreased shortly thereafter, with a much lower value of 88 nM obtained at 3:30 p.m. (Fig. 3a). Though more sampling shortly before or after 1:30 p.m. at this location would presumably provide more detailed information regarding the maximal levels that H_2_O_2_ concentrations could reach at local surface water, our detected value of 186 nM at 1330 h is also a faithful representation, considering that the highest daytime concentration of H_2_O_2_ resulting from photochemical formation was normally reached 1.5 h after the maximum solar irradiance^22^. Extensive measurements of both H_2_O_2_ and solar radiation intensity in the surface water from Water of Leith (Dunedin, New Zealand) over three years by Rusak et al.^23^ suggested that concentrations of H_2_O_2_ in this surface water stream fluctuated roughly concomitant with the intensity of solar UVB radiation with H_2_O_2_ contents higher in summer than in winter as a result of the large summertime increases in solar radiation at sea level in southern New Zealand, particularly with regard to the photochemically important UVB band^74^.

Another repeat measurement of H_2_O_2_ in groundwater at MCP1 was conducted on July 18th with 57 nM H_2_O_2_ found in the morning (8:15 a.m.), as shown in Fig. 3b, which is quite close to the concentration of 59 nM detected at the same location, but in the late afternoon (4:50 p.m.) on July 15th. Though the temporal and spatial patterns of H_2_O_2_ in the groundwater are expected to be affected by a variety of processes, including trace metal mediated redox reactions, the oxidation of reduced organic moieties present in dissolved or soil organic matter^30,55^ and the biologically-mediated generation and decomposition of H_2_O_2_, such nearly constant concentrations observed at MCP1 could also indicate there might be a stable mechanism governing H_2_O_2_ patterns at this location. Indeed, it has been previously shown that the redox gradients along the MCP transect at East River watershed are relatively stable and unresponsive to minor water-level fluctuations (but do respond to storm events and shift on seasonal time scales instead of diel cycles)^32^. Therefore, in this study, we suggest that there is a relatively stable balance between H_2_O_2_ production and consumption at this location with our detected profiles of H_2_O_2_ here more likely a result of a sustained balance between various sources and sinks in the vicinity (i.e., steady-state concentrations). While we cannot completely rule out the possibility that our observed H_2_O_2_ in groundwater at MCP1 was originated from H_2_O_2_ initially present in the river water^70^ as MCP1 sits very close to the river bank and is strongly influenced by surface water recharge, considering the very low concentration of H_2_O_2_ (~ 14 nM) observed in surface river water adjacent to MCP1 at 7:30 a.m. on the same day (Fig. 3a), this is unlikely to be the major contributor of the much elevated H_2_O_2_ (57 nM) detected in groundwater at MCP1 after less than 1 h, indicating that the H_2_O_2_ profiles observed here were a result of active production in the vicinity of the sampling locations.

In order to better probe the pervasiveness of H_2_O_2_ in East River sediments, the measurment of H_2_O_2_ was also carried out at several other locations where piezometric observation wells have been previously installed. Figure 3b presents the various concentrations of H_2_O_2_ (range from 11 to 57 nM) detected in groundwater at a few other locations of the East River aquifer. As shown in Fig. 1, Meander O is a cutoff meander in which the old riverbed channel is seasonally inundated by water during spring snowmelt and does not contribute substantially to the main flow of the river during dry periods^46^. However, the progressive evolution of meander cut-off and oxbow lake formation is expected to dramatically alter the redox gradients within the meander soils/sediments, changing biogeochemical functioning while creating a new biogeochemically distinct feature of an oxbow lake/deposit. Measurements in groundwater close to the new river channel of Meander O (the “neck”) found detectable levels of H_2_O_2_ with 11 nM and 38 nM observed at locations MO3 and MO4, respectively (Fig. 3b). The slightly lower concentrations of H_2_O_2_ at Meander O might be correlated to the lower concentrations of extractable metals found in such seasonally inundated locations than regions that remain unsaturated for most of the year^46^. Alternatively, it’s worth mentioning that MO4 located in a relatively permanent wetland section of the oxbow in the former river channel with high content of aged organic carbon^75^ that could also contribute to the cycling of ROS. Another sample taken at the Mancos Shale seep (approximately 150 m uphill from the oxbow lake of Meander O, Fig. 1) resulted in 52 nM H_2_O_2_ being detected at this location along with a high Fe(II) concentration (~ 12 mg/L) and very little DO (~ 0.4 mg/L), indicating the existence of H_2_O_2_ under an extremely reducing condition. Unlike the sampling locations in the floodplain, the seep location is not influenced by hyporheic exchange, and likely represents deeper and older groundwater. While the quick consumption of DO by Fe(II) could contribute to the observed H_2_O_2_ at this location (Eqs. 2–4), previous studies have indicated that transitional metal species should mainly act as H_2_O_2_ consumers (Eq. 5) under most reducing conditions^28^.

### Environmental implications

As a central interface connecting the stream and groundwater ecosystems, hyporheic zone has been recently identified as a disproportionately important component of the watershed reactor due to its transitional chemical and biological characteristics and distinct ecological functions^33,76^. The oscillating redox conditions, constant biogeochemical exchanges and intensive microbial activities occurring at various spatial and temporal scales in the hyporheic flow create ideal conditions for ROS production. This study represented the field measurement of H_2_O_2_ both in an intra-meander hyporheic zone and in surface water at East River floodplain, CO. Collectively, our results demonstrate that H_2_O_2_ concentrations in the intra-meander transect closely follow the distribution of local redox gradients, ranging from 6 nM detected at the most reduced region to ~ 80 nM at locally oxygen-rich area. There are many possible mechanisms contributing to such H_2_O_2_ patterns, including dark biological production and consumption, and metal-mediated redox interactions between various important elements driven by fluctuating redox conditions. Measurements in surface water revealed typical diel changes of H_2_O_2_ concentrations with a maxima of 186 nM detected at 1:30 pm, lagging the maximum solar irradiance by ∼ 1.5 h, indicating the essential contribution from sunlight-driven photochemical and phototrophic processes. A recent field study on the organic carbon in sediments of this floodplain reported that 23–34% of sediment organic carbon is derived from shale, another large fraction of the Earth's total carbon stocks, demonstrating continuous disturbing of the persistence of aged carbon and its active cycling into riverine systems^75^.

Our observation of the pervasiveness of H_2_O_2_ across the whole intrameander transect suggests that the entire floodplain area defined by the meandering river has the potential to sustain substantial ROS concentrations. For instance, a recent estimate on the volume of groundwater associated with subterranean estuary emission suggested that approximately 6.0 × 10^10^ kg of water exchanged between oxic and anoxic conditions per day for the entire South Carolina coastline, which implied a potential ROS flux of up to 1.5 × 10^7^ mol day^–1^, comparable in magnitude to photochemical sources of ROS in surface waters^11^. Although the generation and consumption of ROS will largely depend on the local redox matrix and microbial activity, such sustained ROS behaviour under rapidly oscillating redox conditions represents a previously under-appreciated source of oxidation that drives biogeochemical processes, such as metal bioavailability and carbon cycling.
